# Estimated Cost-effectiveness of Atezolizumab Plus Cobimetinib and Vemurafenib for Treatment of *BRAF V600* Variation Metastatic Melanoma

**DOI:** 10.1001/jamanetworkopen.2021.32262

**Published:** 2021-11-11

**Authors:** Chao Cai, Ismaeel Yunusa, Ahmad Tarhini

**Affiliations:** 1 Department of Clinical Pharmacy and Outcomes Sciences, University of South Carolina, Columbia; 2 Department of Cutaneous Oncology, Moffitt Cancer Center & Research Institute, Tampa, Florida; 3 Department of Immunology, Moffitt Cancer Center & Research Institute, Tampa, Florida; 4 Department of Oncologic Sciences, Morsani College of Medicine, University of South Florida, Tampa

## Abstract

**Importance:**

In the IMspire150 trial, triplet treatment with atezolizumab and vemurafenib plus cobimetinib significantly improved progression-free survival (PFS) compared with vemurafenib plus cobimetinib alone for treatment of *BRAF V600* variation metastatic melanoma. However, considering high cost of this combination, it is unclear if the incremental cost is worth the additional survival benefit.

**Objective:**

To evaluate the cost-effectiveness of atezolizumab and vemurafenib plus cobimetinib vs vemurafenib plus cobimetinib alone in patients with newly diagnosed unresectable *BRAF V600* variation metastatic melanoma from the US health care perspective.

**Design, Setting, and Participants:**

This economic evaluation study used a 3-state partitioned survival model to assess the cost-effectiveness of the combination of atezolizumab with vemurafenib plus cobimetinib vs vemurafenib plus cobimetinib alone. The observed Kaplan-Meier curves for overall survival and PFS were digitized from the IMspire150 trial (January 2017-April 2018) and the long-term survivals (over a lifetime horizon) beyond the end of the trial were extrapolated using 7 different survival models. The cost and health preference data were collected from a literature review. This study was performed from March 2021 through June 2021.

**Main Outcomes and Measures:**

The outcomes of interest were expected life-years (LYs) gained and quality-adjusted life-years (QALYs), costs, and incremental cost-effectiveness ratio (ICER), expressed as cost per LYs and per QALYs saved.

**Results:**

Adding atezolizumab to vemurafenib and cobimetinib provided an additional 3.267 QALYs compared with the doublet regimen of vemurafenib plus cobimetinib, at an ICER of $271 669 per QALY, which is not considered cost-effective at the willingness-to-pay threshold of $150 000 per QALY. However, the scenario analyses found that atezolizumab combined with vemurafenib plus cobimetinib could be cost-effective at 20-year (ICER, $121 432 per QALY) and 30-year ($98 092 per QALY) time horizons when both strategies were stopped after 2 years of treatments, and over a lifetime horizon ($122 220 per QALY) when only immunotherapy with atezolizumab was stopped after 2 years of treatment.

**Conclusions and Relevance:**

These findings suggest that the atezolizumab and vemurafenib plus cobimetinib regimen provides significant survival benefits over vemurafenib plus cobimetinib alone, and a price reduction would be encouraged to maximize the value of its survival gain.

## Key Points

**Question:**

In patients with *BRAF V600* altered metastatic melanoma, are the incremental costs of triplet therapy of atezolizumab, vemurafenib plus cobimetinib vs vemurafenib plus cobimetinib alone cost-effective for the survival gains?

**Findings:**

In this economic evaluation, the triplet combination therapy was not cost-effective if immunotherapy was continued over a lifetime horizon at a willingness-to-pay threshold of $150 000 per quality-adjusted life-year. However, in real-world practice, in which immunotherapies are often stopped after 2 years, adding atezolizumab to vemurafenib plus cobimetinib could be cost-effective.

**Meaning:**

These findings suggest that atezolizumab and vemurafenib plus cobimetinib regimen provides significant survival benefits over vemurafenib plus cobimetinib alone, and a price reduction would be encouraged to maximize the value of its survival gain.

## Teaser

This economic evaluation assesses the cost-effectiveness of atezolizumab and vemurafenib plus cobimetinib in patients with metastatic melanoma from the US health care perspective

## Introduction

Metastatic melanoma is the most aggressive skin cancer and carries the worst prognosis.^1,2^ Approximately 50% of patients with cutaneous melanoma have tumors that are *BRAF V600* variation positive.^3^ In recent years, therapeutic interventions for advanced melanoma have expanded to include immunotherapies and targeted therapies as 2 main classes of drugs.^4^ Targeted therapy is associated with high but relatively short-lived objective response rates,^3^ while immunotherapy provides more durable responses with relatively lower response rates.^5^ Given the complementary clinical characteristics of these 2 main classes of drugs and a potential for achieving long-term survival, the treatment of metastatic melanoma has been revolutionized by developments in combining targeted therapy with immunotherapy.^4,6,7^

On July 30, 2020, the Food and Drug Administration (FDA) approved atezolizumab (programmed cell death ligand 1 inhibitor) plus vemurafenib (BRAF inhibitor) and cobimetinib (MEK inhibitor) for the treatment of patients with unresectable metastatic *BRAF V600* variation melanoma based on the findings from the pivotal phase 3 IMspire150 trial. This trial had a median (IQR) follow-up of 18.9 (10.4-23.8) months, and the median progression-free survival (PFS) was 15.1 months in the combination therapy arm of atezolizumab plus vemurafenib and cobimetinib vs 10.6 months in the doublet therapy arm with vemurafenib and cobimetinib (hazard ratio [HR], 0.78, 95% CI, 0.63-0.97; *P* = .03), which met the primary PFS end point and provided a high-level of evidence for prolonged PFS.^8^ The objective of this study is to estimate the long-term survival and evaluate the cost-effectiveness of atezolizumab, vemurafenib, and cobimetinib compared with vemurafenib plus cobimetinib alone as first-line treatment for previously untreated patients with unresectable or metastatic *BRAF V600* variation melanoma.

## Methods

### Analytic Overview

This study was exempted from institutional review board approval and informed consent by the University of South Carolina because this is not human participant research. We conducted this study based on recommendations of the US Second Panel on Cost-Effectiveness in Health and Medicine.^9^ The reporting of the study followed the Consolidated Health Economic Evaluation Reporting Standards (CHEERS) reporting guideline.

### Data Source

As the individual patient data for IMspire150 is not publicly available yet, the Kaplan-Meier curves for overall survival (OS) and PFS from the trial were first digitized using WebPlotDigitizer^10^ digitizing software version 4.4 and the algorithms by Guyot et al^11^ and Rakap et al^12^ to impute patient-level time-to-event data (eFigure 1 and eFigure 2 in the Supplement). The data points (x-axis and y-axis coordinates) from the digitized copies of the published survival curves were extracted,^10^ and then the number of events, the number of patients at risk at various time points, and maximum likelihood functions were used to estimate the underlying individual patient data with corresponding time as well as an event indicator.^11,13,14^

From the IMspire150 trial, we can see that the time to progression survival estimates from the Kaplan-Meier curves for triplet combination therapy group and doublet targeted therapy group did not drop to 0 at the end of the trial. For those who had not experienced the primary end point (disease progression) by the trial end, their survival times were censored. By the cutoff date (October 11, 2019), there was still a proportion of patients (approximately 25%) who were censored, suggesting a potential long-term survival benefit beyond the trial time horizon.

### Statistical Analysis

#### Long-term Survival Modeling

We used 7 different survival models to extrapolate survival from the published PFS and OS curves. These included 3 standard parametric survival models (log-normal, log-logistic, and generalized gamma), 3 mixture cure models (MCMs) to account for the potential cure rate in the population (log-normal MCM, log-logistic MCM, and generalized gamma MCM), and 1 flexible survival regression using the Royston/Parmar spline model. We extrapolated the trial survival curves over different time horizons (5, 10, 15, 20, and 30 years) to fully capture the lifetime incremental benefit associated with the newly FDA-approved first-line triplet combination therapy.^14,15,16,17^ The estimated hazard rates and long-term survival outcomes from 7 different survival models are shown in eFigure 3 in the Supplement. The estimated parameters and Akaike information criterion goodness-of-fit statistic values^13^ from each survival model are provided in eTable 1 in the Supplement. The best fit survival model was selected by visually inspecting all the fitted curves and the lowest Akaike information criterion value.^13^

#### Cost-effectiveness Model Construction

**Figure 1.**
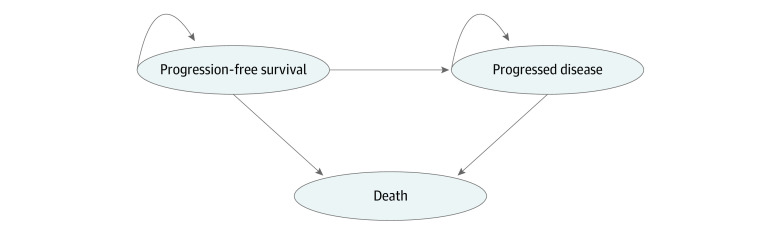
Partitioned Survival Model with 3 Health States Each oval shape represents a health-state. Patients can remain in progression-free or progressed disease health state, move from progression-free to progressed disease health state, or move from either state to the death state.

We evaluated the cost-effectiveness of triplet combination therapy of atezolizumab with vemurafenib plus cobimetinib in patients with histologically confirmed, previously untreated metastatic *BRAF V600* variation melanoma. This population is consistent with the population evaluated in the IMspire150 trial. The cost-effectiveness analysis used a 3-state partitioned survival model.^18,19^ As shown in Figure 1, the health-states in the partitioned survival model included PFS, postprogression survival (PPS), and death. Patients were assumed to start in the PFS health state, in which they may experience disease progression, enter the PPS state, or die and enter the death state. Patients who were alive were partitioned according to progression status (ie, progression-free or postprogression). Over time, membership in the states was determined by survival curves for PFS and OS. The survival curve for PFS provided the proportion of patients alive who were in the PFS health state over time. Membership in the death state was calculated as the complement of the OS curve. Membership in the PPS state was calculated as the difference between PFS and OS. The probabilities of membership in the death health state over time were estimated as 1 − probability of OS, which was estimated using a similar approach as that used for PFS. Membership in the PPS state was calculated as the difference between PFS and OS.

#### Cost and Utility Inputs

The model’s cost inputs for drugs (eTable 2 in the Supplement) were obtained based on mean wholesale price from the Red Book (Truven Health Analytics). Cost of treating progression-free and progressed disease and utility inputs were obtained from the published literature (eTable 3 and eTable 4 in the Supplement).^20,21,22,23,24,25^

#### Outcomes

The model had a periodicity of 1-month cycle length. Three categories of outcome measures were calculated by the model: measures of effectiveness (life-years [LYs] gained and quality-adjusted life-years [QALYs]), costs, and cost-effectiveness (incremental cost-effectiveness ratios [ICERs]), expressed as cost per LY and per QALY saved). The model calculated total expected costs for each strategy by summing across all the cost categories. Costs, LYs, and QALYs, were discounted on an annual basis of 3% beginning 1 year after therapy initiation.^9^

#### Base Case Analysis

We calculated the base case ICER for cost-effectiveness expressed as atezolizumab and vemurafenib with cobimetinib vs vemurafenib with cobimetinib alone. Of note, in the US, there is no consensus on a willingness-to-pay (WTP) threshold value. However, Neumann et al^26^ recommended a threshold of $100 000 or $150 000 per QALY for a disease like melanoma that is expensive to treat in situations in which there is no explicit resource constraint. In this study, the WTP threshold was determined at an ICER of less than $150 000/QALY over a patient’s lifetime (30 years),^21^ consistent with other studies of treatment of advanced melanoma. A lifetime horizon was necessary for the base case analysis, given the need to account for all important costs and health outcomes.^27,28,29^

#### Scenario and Sensitivity Analyses

In practice, the length of immunotherapy could be 2 years, as supported by the KEYNOTE-006 phase 3 trial for pembrolizumab in metastatic melanoma,^30^ so the costs were extrapolated with different treatment capping scenarios modeled as both strategies were stopped after 2 years of treatments or only immunotherapy was stopped after 2 years of treatment. Economic outcomes were estimated under time horizons of 5, 10, 15, 20, and 30 years to examine the robustness of the estimated long-term survival and cost-effectiveness model. To examine the robustness of our findings and characterize joint parameter uncertainty in all model inputs, deterministic sensitivity analysis (DSA) and probabilistic sensitivity analysis (PSA) were also conducted. Parameters included in the DSA were varied by an SD of 25% of base case estimates. The PSAs were conducted by sampling from estimated probability distributions of model parameters (eTable 5 in the Supplement) with 1000 simulated data sets. For each simulation, expected total costs^31^ (sum of expected cost for PFS and PPS health states) and QALYs were calculated, along with the differences between strategies in expected costs and QALYs. Cost-effectiveness acceptability curves were also constructed.^32^

All modeling analyses were performed in R statistical software version 4.1.0 (R Project for Statistical Computing) and the hēRo3 platform, a web-based, open-source health economic modeling platform (Health Economics in R Online). This study was performed from March 2021 through June 2021.

## Results

### Base-Case Analysis

Based on extrapolated survival data from the IMspire150 trial, treating patients with newly diagnosed unresectable locally advanced or metastatic *BRAF V600* variation melanoma with vemurafenib plus cobimetinib was associated with a lifetime expected cost of $1 205 321 for an expected gain of 5.733 LYs per patient. However, adding atezolizumab to this atezolizumab regimen was associated with a higher expected lifetime cost of $2 092 986 and an expected gain of 7.720 LYs per patient (Table 1). Compared with vemurafenib plus cobimetinib therapy, atezolizumab and vemurafenib plus cobimetinib treatment provided an additional 3.267 QALYs, with an incremental cost of $887 665, which was associated with a lifetime ICER of $271 669 per QALY, which could be interpreted as not cost-effective at a WTP threshold of $150 000 per QALY gained.

**Table 1.** Base Case Results of the Cost-effectiveness Analysis Over a 30-Year Time Horizon

Expected outcome	Vemurafenib + cobimetinib	Atezolizumab + vemurafenib + cobimetinib	Incremental
Cost, $	1 205 321	2 092 986	887 665
Life-years	5.733	11.714	5.980
QALY	4.453	7.720	3.267
ICER, cost per QALY, $	NA	NA	271 669

Abbreviations: ICER, incremental cost-effectiveness ratio; NA, not applicable; QALY, quality-adjusted life-year.

### Scenario and Sensitivity Analyses

In a scenario in which both strategies were stopped after 2 years of treatment, atezolizumab in combination with vemurafenib plus cobimetinib could be cost-effective at 20-year (ICER, $121 432 per QALY) and 30-year ($98 092 per QALY) time horizons. Expected costs, LYs, and QALYs increased as the time horizon increased while ICER decreased (Table 2). When only immunotherapy with atezolizumab was stopped after 2 years of treatment, we found that it would be cost-effective over a lifetime horizon ($122 220 per QALY).

**Table 2.** Results of the Scenario Analyses

Measure	Time horizon, y				
	5	10	15	20	30
**Immunotherapy and targeted therapy capped at 2 y**					
Total cost, $					
Atezolizumab + vemurafenib + cobimetinib	569 044	596 563	621 439	643 478	679 666
Vemurafenib + cobimetinib	334 644	343 937	349 244	353 206	359 160
Incremental	234 400	252 626	272 195	290 272	320 505
Discounted total life-years					
Atezolizumab + vemurafenib + cobimetinib	3.279	5.521	7.430	9.074	11.714
Vemurafenib + cobimetinib	2.729	3.706	4.370	4.903	5.733
Incremental	0.549	1.815	3.060	4.171	5.980
Discounted total quality-adjusted life-years					
Atezolizumab + vemurafenib + cobimetinib	2.462	3.932	5.129	6.135	7.720
Vemurafenib + cobimetinib	2.005	2.750	3.295	3.744	4.453
Incremental	0.456	1.182	1.834	2.390	3.267
ICER					
$/Life-years	426 587	139 187	88 947	69 595	53 593
$/Quality-adjusted life-years	513 544	213 645	148 448	121 432	98 092
**Immunotherapy capped at 2 y**					
Total cost $					
Atezolizumab + vemurafenib + cobimetinib	810 639	1 076 835	1 261 279	1 396 573	1 604 662
Vemurafenib + cobimetinib	487 124	681 854	843 866	982 723	1 205 321
Incremental	323 515	394 981	417 413	419 235	399 341
Discounted total life-years					
Atezolizumab + vemurafenib + cobimetinib	3.279	5.521	7.430	9.074	11.714
Vemurafenib + cobimetinib	2.729	3.706	4.370	4.903	5.733
Incremental	0.549	1.815	3.060	4.171	5.980
Discounted total quality-adjusted life-years					
Atezolizumab + vemurafenib + cobimetinib	2.462	3.932	5.129	6.135	7.720
Vemurafenib + cobimetinib	2.005	2.750	3.295	3.744	4.453
Incremental	0.456	1.182	1.834	2.390	3.267
ICER					
$/Life-years	588 768	217 619	136 401	100 515	66 775
$/Quality-adjusted life-years	708 784	334 035	227 646	175 383	122 220

Abbreviation: ICER, incremental cost-effectiveness ratio.

**Figure 2.**
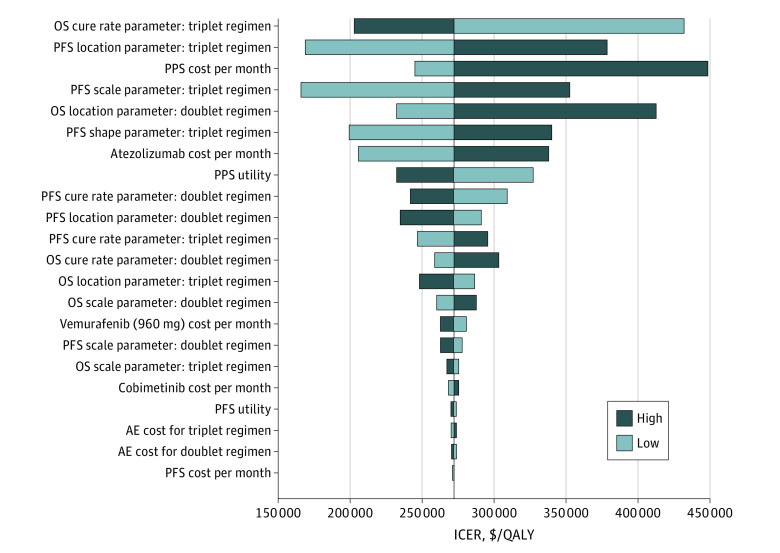
Results of Deterministic Sensitivity Analysis The central black line represents the base-case incremental cost-effectiveness ratio. The bars are arranged in order, with the widest bar (potentially the most influential to incremental cost-effectiveness ratio) at the top and the narrowest bar at the bottom. AE indicates adverse events; doublet regimen, vemurafenib plus cobimetinib; ICER, incremental cost-effectiveness ratio; OS, overall survival; PFS, progression-free survival; PPS, postprogression survival; QALY, quality-adjusted life-year; and triplet regimen, atezolizumab with vemurafenib plus cobimetinib.

**Figure 3.**
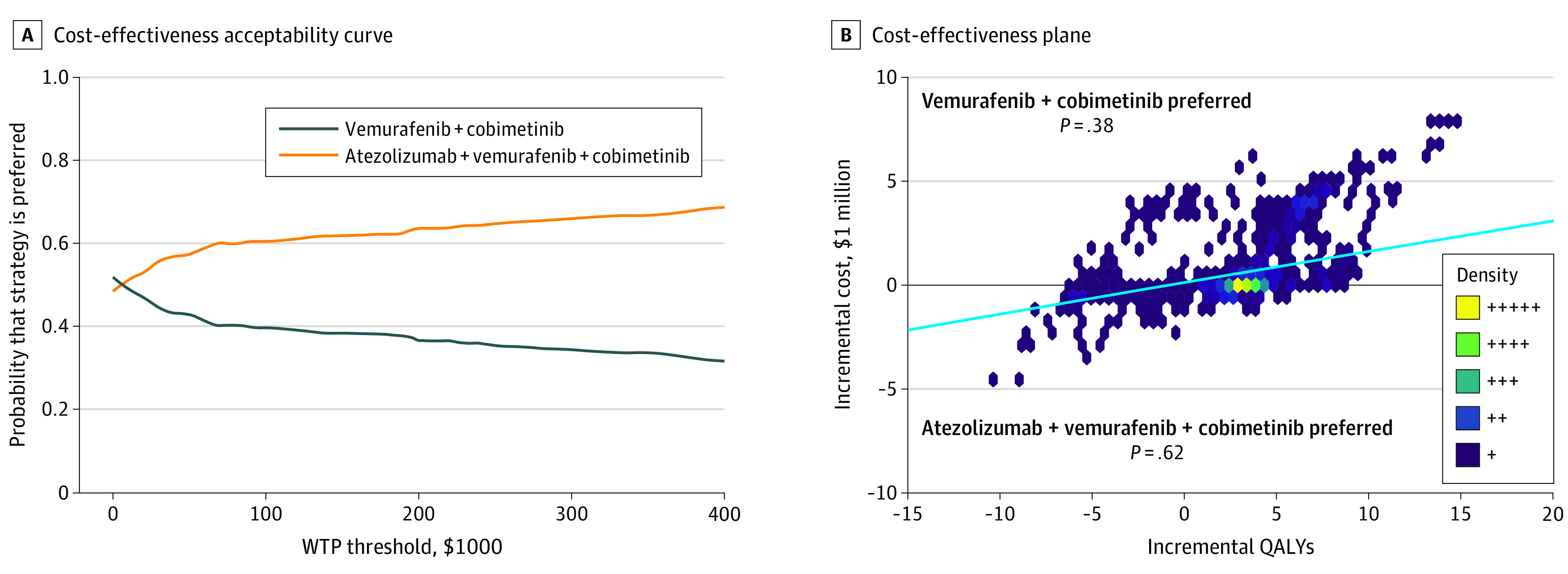
Results of Probabilistic Sensitivity Analysis A, The cost-effectiveness acceptability curve shows the probabilities of being cost-effective at different willingness-to-pay (WTP) thresholds for atezolizumab and vemurafenib plus cobimetinib compared to vemurafenib plus cobimetinib. B, Each dot represents 1 simulation run, and there is a total of 1000 iterations. The solid blue line indicates WTP thresholds per quality-adjusted life-year (QALY). The number of dots below a specific line represent the probability for atezolizumab and vemurafenib plus cobimetinib to be cost-effective at the $150 000 per QALY WTP threshold.

The results of DSA suggest that OS cure rate parameter for triplet regimen had the greatest influence on the ICER, followed by PFS location parameters for triplet regimen, PPS monthly cost, PFS scale parameters for triplet regimen, OS location parameters for doublet regimen, PFS shape parameters for triplet regimen, atezolizumab monthly cost, and PPS utility (Figure 2). The PSA suggested that, given the current data, the probability of vemurafenib plus cobimetinib alone being cost-effective compared with atezolizumab and vemurafenib plus cobimetinib was 38.3% at a WTP of $150 000 per QALY (Figure 3).

## Discussion

This economic evaluation is the first study of a combination of an immunotherapeutic agent with a doublet targeted therapy in patients with advanced melanoma in the United States, to our knowledge. The FDA approval of the atezolizumab plus vemurafenib and cobimetinib triplet therapy for patients with advanced melanoma provided an effective treatment strategy with improved survival benefits over the targeted therapy doublet but at an additional cost. Therefore, examining the cost-effectiveness of this treatment to determine if the incremental cost is worth the additional survival benefit is necessary for the judicious use of scarce health care resources. Based on the results of the IMspire150 trial, this study found that, over a lifetime horizon, atezolizumab in combination with vemurafenib plus cobimetinib would not be cost-effective at the WTP threshold of $150 000 per QALY for treating patients with newly diagnosed unresectable locally advanced or metastatic *BRAF V600* variation melanoma. This finding implies that, for triplet therapy with atezolizumab and vemurafenib plus cobimetinib to be cost-effective, the price of this regimen needs to be substantially reduced for its incremental cost to be worth the additional benefit.

This study’s findings are consistent with other cost-effectiveness analyses of the atezolizumab-containing regimen in different malignant diseases, such as non–small cell lung cancer and metastatic hepatocellular carcinoma. The studies consistently found that atezolizumab-containing regimens are not cost-effective, underscoring the need for a substantial price reduction or discount.^33,34,35^ Given that, as evidence suggests, increasing drug costs contribute to inaccessibility, a substantial reduction in price or discount would enable patients to get this regimen with proven survival benefit compared with targeted therapy alone.^36^ Additionally, considerable reduction in price or discount of the triplet regimen in patients with advanced melanoma in the US would be consistent with the recommendation of the National Institute for Health and Care Excellence in the UK, in which it found that vemurafenib plus cobimetinib was not cost-effective.^37^

In a scenario analysis, we found that atezolizumab in combination with vemurafenib plus cobimetinib could be cost-effective if oncologists stop immunotherapy at 2 years. Although there is no consensus on the optimal duration of immunotherapy in patients with melanoma, these findings suggest that the incremental cost of the triplet strategy with atezolizumab combined with vemurafenib plus cobimetinib when physicians stop treatment at 2 years would be worth the additional clinical benefit. It is important to note that the KEYNOTE-006 phase 3 trial leading to the approval of pembrolizumab in metastatic melanoma stopped pembrolizumab at 2 years, a strategy that was further supported by long-term follow up from this trial.^30^ In clinical practice, it is safe to assume that patients and treating oncologists would be willing to stop systemic immunotherapy after 2 years in the absence of disease progression and of data supporting the need for lifelong treatment continuation.

A 2020 systematic review by Gorry et al^38^ that examined the cost-effectiveness of pharmacological treatments of advanced melanoma found that BRAF- and MEK-inhibitor doublet targeted therapy combinations, such as vemurafenib plus cobimetinib, compared with a BRAF monotherapy or chemotherapy, were consistently not cost-effective. This might be justified by the high level of uncertainty that vemurafenib plus cobimetinib combination from our PSA results, in which we found that the probability of vemurafenib plus cobimetinib combination being cost-effective was only 38.3%. The systematic review also found that monotherapy with programmed cell death immunotherapies, such as pembrolizumab and nivolumab, were consistently cost-effective compared with ipilimumab, a CTLA-4 inhibitor.^38^ Owing to its long-lasting immunotherapeutic effect even when stopped after 24 months, the addition of atezolizumab to the vemurafenib plus cobimetinib regimen might explain why we found that the triplet strategy was cost-effective over a lifetime horizon if physicians only stop treatment with either both (immunotherapy and targeted therapy) or only immunotherapy with atezolizumab after 2 years.

Unlike previous economic evaluations of immunotherapy or targeted treatments of patients with advanced melanoma, this cost-effectiveness analysis accounted for the proportion of the patients cured by using MCM, assuming patients who were censored at the end of the follow-up in the IMspire150 trial are potential long-term survivors. Ignoring the proportion of the cured patients by using the standard survival models only, as is the case with previous studies, will underestimate the long-term clinical and economic outcomes by assuming the same mortality rate for all patients. This has been demonstrated by Whittington et al^17^ in an economic evaluation in which standard parametric approaches resulted in a smaller incremental gain in QALYs and inflating ICERs, while the MCM approach led to a more accurate cost-effectiveness estimate. Finally, our findings may have implications related to future triplet combination therapy regimens currently in development in melanoma and other malignant neoplasms, for which cost-effectiveness analyses will become increasingly essential in the face of already stretched health care resources.

### Limitations

This study has some limitations. First, the long-term survival and cost-effectiveness model essentially relied on the validity and generalizability of the IMspire150 trial, which was the first phase 3 randomized clinical trial to investigate a triplet immuno-targeted therapy regimen in patients with previously untreated metastatic or unresectable locally advanced melanoma with a *BRAF V600* variation. Any biases within the trial may have affected the cost and effectiveness estimates. Second, the patient-level data were not available to the public. Therefore, the survival analysis was based on digitized pseudo trial data. Third, the percentage of the population that is assumed to be cured requires empirical confirmation, and a longer follow-up is needed to substantiate the survival estimates. As a newly FDA-approved combination regimen of immunotherapy plus targeted therapy, long-term outcomes are unknown. We also assumed survival estimates would remain valid if the immunotherapy ended after 2 years. Additionally, this cost-effectiveness analysis only compared 2 treatment strategies evaluated in the IMspire150 trial. Future cost-effectiveness analyses should compare all other treatment options following a network meta-analysis that will generate relative HRs comparing the treatment alternatives directly and/or indirectly.

## Conclusions

In this economic evaluation, adding atezolizumab to vemurafenib plus cobimetinib was associated with survival and quality-of-life improvement but was not cost-effective at a WTP value of $150 000 per QALY over a lifetime horizon compared with vemurafenib plus cobimetinib alone for patients with newly diagnosed advanced or metastatic BRAF variation melanoma from a US health care perspective. Thus, there is a need for a substantial reduction in price for this regimen, which may be needed for its incremental cost to be worth the additional benefit. However, in real-world practice, in which immunotherapies are often stopped after 2 years of treatment, adding atezolizumab to vemurafenib plus cobimetinib could be cost-effective.

## Notes

Supplement. **eFigure 1.** Replicated PFS Curves of Atezolizumab Plus Vemurafenib Plus Cobimetinib vs Vemurafenib Plus Cobimetinib From IMspire150 Trial **eFigure 2.** Replicated OS Curves of Atezolizumab Plus Vemurafenib Plus Cobimetinib vs Vemurafenib Plus Cobimetinib From IMspire150 Trial **eFigure 3.** Estimated Hazard Rates and Long-term Survival Outcomes **eTable 1.** Estimated Parameters and Goodness-of-Fit AIC Values From Each Survival Model **eTable 2.** Drug Dose and Costs Inputs **eTable 3.** Utilities Inputs **eTable 4.** Adverse Events Rates and Costs Inputs **eTable 5.** Parameter Input in Probabilistic Sensitivity Analysis
eReferences

## References

[1] Yu C, Liu X, Yang J, . Combination of immunotherapy with targeted therapy: theory and practice in metastatic melanoma. Front Immunol. 2019;10:990. doi:10.3389/fimmu.2019.00990 31134073 PMC6513976

[2] Blank CU, Hooijkaas AI, Haanen JB, Schumacher TN. Combination of targeted therapy and immunotherapy in melanoma. Cancer Immunol Immunother. 2011;60(10):1359-1371. doi:10.1007/s00262-011-1079-2 21847631 PMC11028983

[3] Kakadia S, Yarlagadda N, Awad R, . Mechanisms of resistance to BRAF and MEK inhibitors and clinical update of US Food and Drug Administration-approved targeted therapy in advanced melanoma. Onco Targets Ther. 2018;11:7095-7107. doi:10.2147/OTT.S182721 30410366 PMC6200076

[4] Atkins MB, Tarhini A, Rael M, . Comparative efficacy of combination immunotherapy and targeted therapy in the treatment of *BRAF*-mutant advanced melanoma: a matching-adjusted indirect comparison. Immunotherapy. 2019;11(7):617-629. doi:10.2217/imt-2018-0208 30852924

[5] Khair DO, Bax HJ, Mele S, . Combining immune checkpoint inhibitors: established and emerging targets and strategies to improve outcomes in melanoma. Front Immunol. 2019;10:453. doi:10.3389/fimmu.2019.00453 30941125 PMC6435047

[6] Domingues B, Lopes JM, Soares P, Pópulo H. Melanoma treatment in review. Immunotargets Ther. 2018;7:35-49. doi:10.2147/ITT.S134842 29922629 PMC5995433

[7] Villanueva J, Vultur A, Herlyn M. Resistance to BRAF inhibitors: unraveling mechanisms and future treatment options. Cancer Res. 2011;71(23):7137-7140. doi:10.1158/0008-5472.CAN-11-1243 22131348 PMC3588168

[8] Gutzmer R, Stroyakovskiy D, Gogas H, . Atezolizumab, vemurafenib, and cobimetinib as first-line treatment for unresectable advanced *BRAF V600* mutation-positive melanoma (IMspire150): primary analysis of the randomised, double-blind, placebo-controlled, phase 3 trial. Lancet. 2020;395(10240):1835-1844. doi:10.1016/S0140-6736(20)30934-X 32534646

[9] Sanders GD, Neumann PJ, Basu A, . Recommendations for conduct, methodological practices, and reporting of cost-effectiveness analyses: second panel on cost-effectiveness in health and medicine. JAMA. 2016;316(10):1093-1103. doi:10.1001/jama.2016.12195 27623463

[10] Rohatgi A. WebPlotDigitizer. Accessed October 12, 2021. https://automeris.io/WebPlotDigitizer/

[11] Guyot P, Ades AE, Ouwens MJ, Welton NJ. Enhanced secondary analysis of survival data: reconstructing the data from published Kaplan-Meier survival curves. BMC Med Res Methodol. 2012;12(1):9. doi:10.1186/1471-2288-12-9 22297116 PMC3313891

[12] Rakap S, Rakap S, Evran D, Cig O. Comparative evaluation of the reliability and validity of three data extraction programs: UnGraph, GraphClick, and DigitizeIt. Computers in Human Behavior. 2016;55:159-166. doi:10.1016/j.chb.2015.09.008

[13] Latimer NR. Survival analysis for economic evaluations alongside clinical trials—extrapolation with patient-level data: inconsistencies, limitations, and a practical guide. Med Decis Making. 2013;33(6):743-754. doi:10.1177/0272989X12472398 23341049

[14] Bullement A, Latimer NR, Bell Gorrod H. Survival extrapolation in cancer immunotherapy: a validation-based case study. Value Health. 2019;22(3):276-283. doi:10.1016/j.jval.2018.10.007 30832965

[15] Othus M, Bansal A, Koepl L, Wagner S, Ramsey S. Accounting for cured patients in cost-effectiveness analysis. Value Health. 2017;20(4):705-709. doi:10.1016/j.jval.2016.04.011 28408015

[16] Whittington MD, McQueen RB, Ollendorf DA, . Long-term survival and value of chimeric antigen receptor T-cell therapy for pediatric patients with relapsed or refractory leukemia. JAMA Pediatr. 2018;172(12):1161-1168. doi:10.1001/jamapediatrics.2018.2530 30304407 PMC6583018

[17] Whittington MD, McQueen RB, Ollendorf DA, . Long-term survival and cost-effectiveness associated with axicabtagene ciloleucel vs chemotherapy for treatment of B-cell lymphoma. JAMA Netw Open. 2019;2(2):e190035-e190035. doi:10.1001/jamanetworkopen.2019.0035 30794298 PMC6484589

[18] Gibson EJ, Begum N, Koblbauer I, . Modeling the economic outcomes of immuno-oncology drugs: alternative model frameworks to capture clinical outcomes. Clinicoecon Outcomes Res. 2018;10:139-154. doi:10.2147/CEOR.S144208 29563820 PMC5848668

[19] Woods BS, Sideris E, Palmer S, Latimer N, Soares M. Partitioned survival and state transition models for healthcare decision making in oncology: where are we now? Value Health. 2020;23(12):1613-1621. doi:10.1016/j.jval.2020.08.2094 33248517

[20] Curl P, Vujic I, van ’t Veer LJ, Ortiz-Urda S, Kahn JG. Cost-effectiveness of treatment strategies for *BRAF*-mutated metastatic melanoma. PLoS One. 2014;9(9):e107255. doi:10.1371/journal.pone.0107255 25198196 PMC4157865

[21] Tarhini A, McDermott D, Ambavane A, . Clinical and economic outcomes associated with treatment sequences in patients with *BRAF*-mutant advanced melanoma. Immunotherapy. 2019;11(4):283-295. doi:10.2217/imt-2018-0168 30563395

[22] Stellato D, Gerbasi ME, Ndife B, . Budget impact of dabrafenib and trametinib in combination as adjuvant treatment of *BRAF V600*E/K mutation-positive melanoma from a US commercial payer perspective. J Manag Care Spec Pharm. 2019;25(11):1227-1237. doi:10.18553/jmcp.2019.25.11.1227 31663466 PMC10398148

[23] Wong W, Yim YM, Kim A, . Assessment of costs associated with adverse events in patients with cancer. PLoS One. 2018;13(4):e0196007. doi:10.1371/journal.pone.0196007 29652926 PMC5898735

[24] Barzey V, Atkins MB, Garrison LP, Asukai Y, Kotapati S, Penrod JR. Ipilimumab in 2nd line treatment of patients with advanced melanoma: a cost-effectiveness analysis. J Med Econ. 2013;16(2):202-212. doi:10.3111/13696998.2012.739226 23057750

[25] Arondekar B, Curkendall S, Monberg M, . Economic burden associated with adverse events in patients with metastatic melanoma. J Manag Care Spec Pharm. 2015;21(2):158-164. doi:10.18553/jmcp.2015.21.2.158 25615005 PMC10397691

[26] Neumann PJ, Cohen JT, Weinstein MC. Updating cost-effectiveness—the curious resilience of the $50,000-per-QALY threshold. N Engl J Med. 2014;371(9):796-797. doi:10.1056/NEJMp1405158 25162885

[27] Basu A, Maciejewski ML. Choosing a time horizon in cost and cost-effectiveness analyses. JAMA. 2019;321(11):1096-1097. doi:10.1001/jama.2019.1153 30789668

[28] Kim DD, Wilkinson CL, Pope EF, Chambers JD, Cohen JT, Neumann PJ. The influence of time horizon on results of cost-effectiveness analyses. Expert Rev Pharmacoecon Outcomes Res. 2017;17(6):615-623. doi:10.1080/14737167.2017.1331432 28504026

[29] Caro JJ, Briggs AH, Siebert U, Kuntz KM; ISPOR-SMDM Modeling Good Research Practices Task Force. Modeling good research practices—overview: a report of the ISPOR-SMDM Modeling Good Research Practices Task Force—1. Value Health. 2012;15(6):796-803. doi:10.1016/j.jval.2012.06.012 22999128

[30] Robert C, Schachter J, Long GV, ; KEYNOTE-006 investigators. Pembrolizumab versus ipilimumab in advanced melanoma. N Engl J Med. 2015;372(26):2521-2532. doi:10.1056/NEJMoa1503093 25891173

[31] Miners A. Estimating ‘costs’ for cost-effectiveness analysis. Pharmacoeconomics. 2008;26(9):745-751. doi:10.2165/00019053-200826090-00005 18767895

[32] Briggs A, Sculpher M, Claxton K. Decision Modelling for Health Economic Evaluation. Oxford University Press; 2006.

[33] Criss SD, Mooradian MJ, Watson TR, Gainor JF, Reynolds KL, Kong CY. Cost-effectiveness of atezolizumab combination therapy for first-line treatment of metastatic nonsquamous non–small cell lung cancer in the United States. JAMA Netw Open. 2019;2(9):e1911952-e1911952. doi:10.1001/jamanetworkopen.2019.11952 31553470 PMC6764123

[34] Su D, Wu B, Shi L. Cost-effectiveness of atezolizumab plus bevacizumab vs sorafenib as first-line treatment of unresectable hepatocellular carcinoma. JAMA Netw Open. 2021;4(2):e210037-e210037. doi:10.1001/jamanetworkopen.2021.0037 33625508 PMC7905498

[35] Zhang X, Wang J, Shi J, Jia X, Dang S, Wang W. Cost-effectiveness of atezolizumab plus bevacizumab vs sorafenib for patients with unresectable or metastatic hepatocellular carcinoma. JAMA Netw Open. 2021;4(4):e214846-e214846. doi:10.1001/jamanetworkopen.2021.4846 33825837 PMC8027915

[36] National Academies of Sciences, Engineering, and Medicine. Making Medicines Affordable: A National Imperative. National Academies Press; 2018. 29620830

[37] National Institute for Health and Care Excellence. Final appraisal determination: cobimetinib in combination with vemurafenib for treating unresectable or metastatic *BRAF V600* mutation-positive melanoma. Accessed October 12, 2021. https://www.nice.org.uk/guidance/ta414/documents/final-appraisal-determination-document

[38] Gorry C, McCullagh L, Barry M. Economic evaluation of systemic treatments for advanced melanoma: a systematic review. Value Health. 2020;23(1):52-60. doi:10.1016/j.jval.2019.07.003 31952674

