# Supplementary material for: Estimated Cost-effectiveness of Atezolizumab Plus Cobimetinib and Vemurafenib for Treatment of BRAF V600 Variation Metastatic Melanoma
Source: JAMA Netw Open. 2021 Nov 11;4(11):e2132262. doi: 10.1001/jamanetworkopen.2021.32262 (PMC8586909; doi:10.1001/jamanetworkopen.2021.32262)
Supplement: Supplement. — eFigure 1. Replicated PFS Curves of Atezolizumab Plus Vemurafenib Plus Cobimetinib vs Vemurafenib Plus Cobimetinib From IMspire150 Trial eFigure 2. Replicated OS Curves of Atezolizumab Plus Vemurafenib Plus Cobimetinib vs Vemurafenib Plus Cobimetinib From IMspire150 Trial eFigure 3. Estimated Hazard Rates and Long-term Survival Outcomes eTable 1. Estimated Parameters and Goodness-of-Fit AIC Values From Each Survival Model eTable 2. Drug Dose and Costs Inputs eTable 3. Utilities Inputs eTable 4. Adverse Events Rates and Costs Inputs eTable 5. Parameter Input in Probabilistic Sensitivity Analysis eReferences [file jamanetwopen-e2132262-s001.pdf]

Supplemental Online Content

Cai C, Yunusa I, Tarhini A. Estimated cost-effectiveness of atezolizumab plus cobimetinib and vemurafenib for treatment of *BRAF V600* variation metastatic melanoma. *JAMA Netw Open*. 2021;4(11):e2132262. doi:10.1001/jamanetworkopen.2021.32262

eFigure 1. Replicated PFS Curves of Atezolizumab Plus Vemurafenib Plus Cobimetinib vs Vemurafenib Plus Cobimetinib From IMspire150 Trial

eFigure 2. Replicated OS Curves of Atezolizumab Plus Vemurafenib Plus Cobimetinib vs Vemurafenib Plus Cobimetinib From IMspire150 Trial

eFigure 3. Estimated Hazard Rates and Long-term Survival Outcomes

eTable 1. Estimated Parameters and Goodness-of-Fit AIC Values From Each Survival Model

eTable 2. Drug Dose and Costs Inputs

eTable 3. Utilities Inputs

eTable 4. Adverse Events Rates and Costs Inputs

eTable 5. Parameter Input in Probabilistic Sensitivity Analysis

eReferences

This supplemental material has been provided by the authors to give readers additional information about their work.

© 2021 Cai C et al. *JAMA Network Open.*

eFigure 1. Replicated PFS Curves of Atezolizumab Plus Vemurafenib Plus Cobimetinib vs Vemurafenib Plus Cobimetinib From IMspire150 Trial

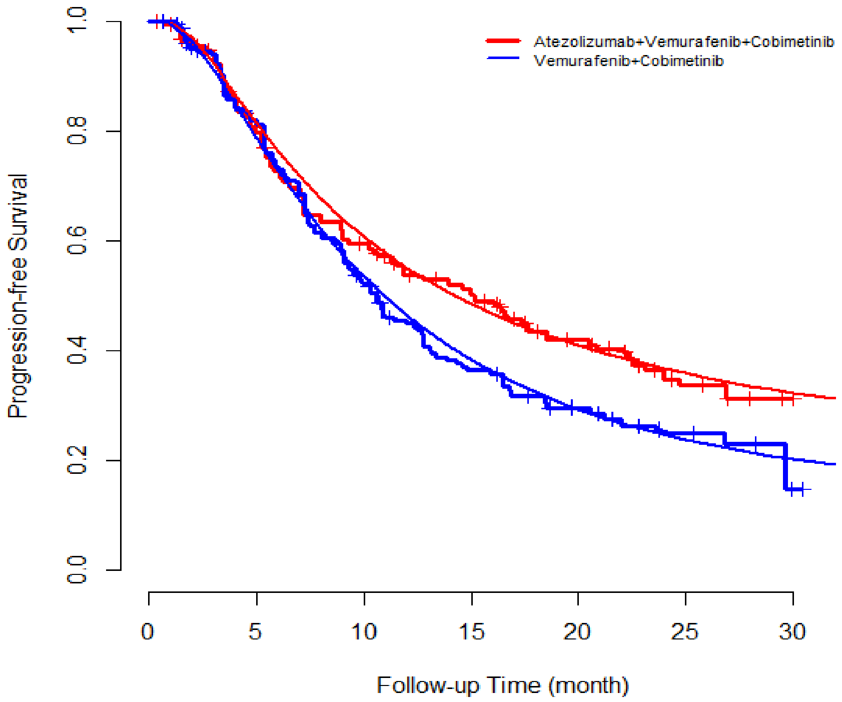

eFigure 2. Replicated OS Curves of Atezolizumab Plus Vemurafenib Plus Cobimetinib vs Vemurafenib Plus Cobimetinib From IMspire150 Trial

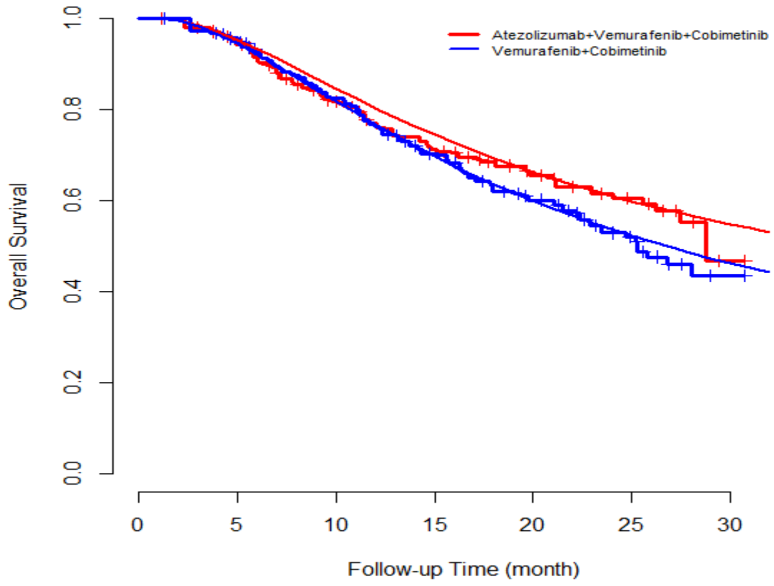

© 2021 Cai C et al. *JAMA Network Open.*

eFigure 3. Estimated Hazard Rates and Long-term Survival Outcomes

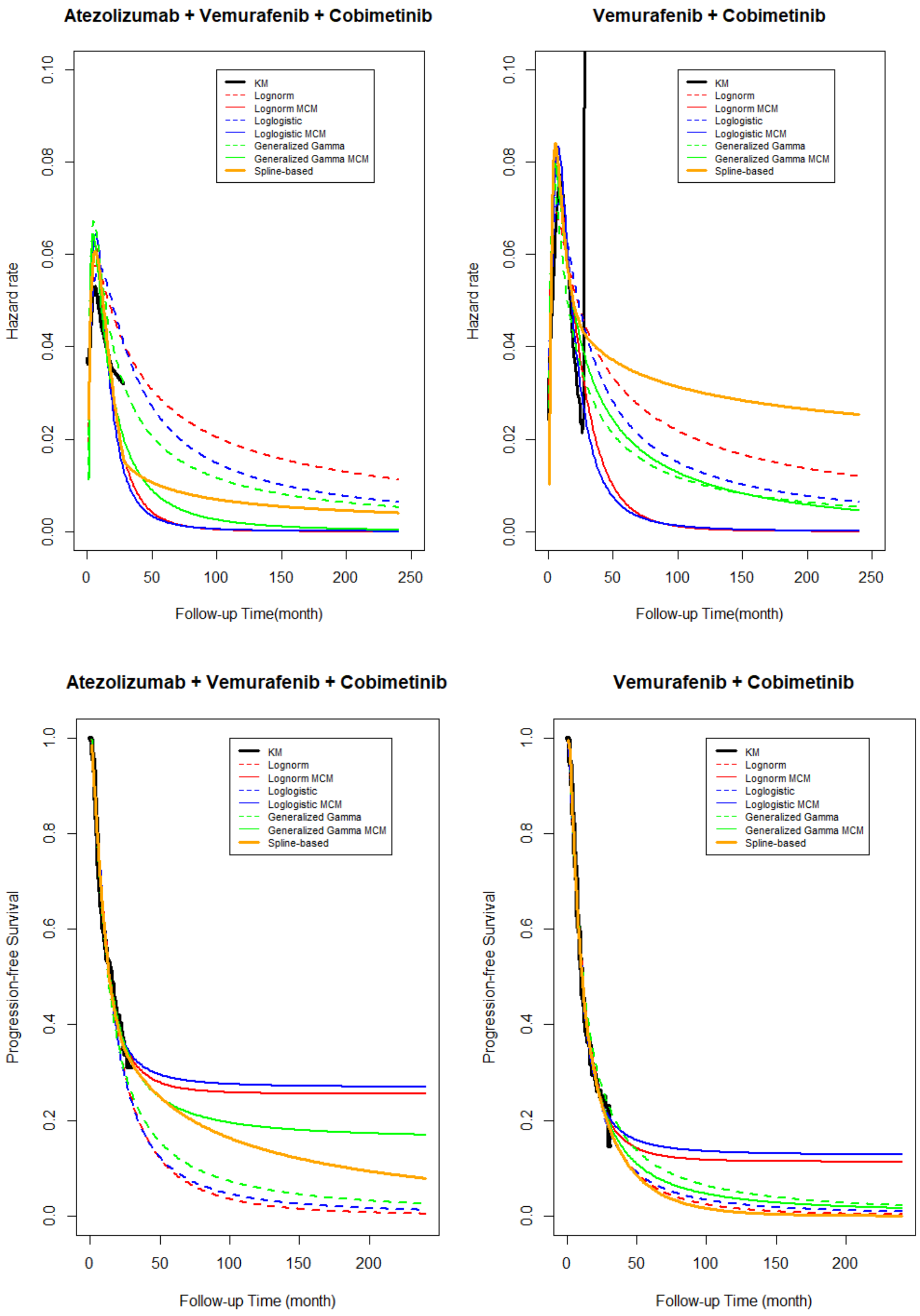

eFigure 3 shows the KM curve and fitted curves using 7 different survival models for triplet combination therapy of Atezolizumab plus vemurafenib plus cobimetinib versus doublet targeted therapy of vemurafenib plus cobimetinib. In both plots, the KM curve is shown as a black solid line, the three standard parametric models without a cure fraction are shown as dotted lines, the three mixture cure models are shown as solid lines (Log-normal in red, log-logistic in blue and generalized gamma in green), and the flexible survival with Royston/Parmar spline model is shown as orange solid line.

© 2021 Cai C et al. *JAMA Network Open.*

eTable 1. Estimated Parameters and Goodness-of-Fit AIC Values From Each Survival Model

Strategy	Distribution	Parameter	PFS			OS		
			Estimate	SE	AIC	Estimate	SE	AIC
**Atezolizumab +Vemurafenib +Cobimetinib**	Log-normal	meanlog	2.69	0.09	1216.92	3.45	0.11	883.84
		sdlog	1.20	0.07		1.17	0.10	
	Log-logistic	shape	1.39	0.09	1225.32	1.49	0.13	889.68
		scale	14.45	1.23		30.35	3.02	
	Generalized Gamma	mu	2.25	0.19	1212.34	2.97	0.31	882.19
		sigma	1.26	0.08		1.39	0.11	
		Q	-0.88	0.32		-1.17	0.60	
	Log-logistic MCM	theta	0.33	NA	1181.30	0.60	NA	839.44
		shape	1.89	0.21		2.22	0.41	
		scale	8.80	1.16		13.55	3.15	
	Lognormal MCM	theta	0.31	NA	1177.10	0.55	NA	837.06
		meanlog	2.22	0.16		2.72	0.33	
		sdlog	0.92	0.10		0.82	0.17	
	Generalized Gamma MCM	theta	0.06	NA	1176.76	0.01	NA	837.62
		mu	2.18	0.25		3.00	0.46	
		sigma	1.22	0.35		1.52	0.34	
		Q	-1.11	0.94		-1.73	1.02	
	Spline-based Model	gamma0	-4.33	0.36	1219.36	-5.87	0.60	887.62
		gamma1	1.87	0.20		1.90	0.27	
		gamma2	-1.23	0.26		-4.12	1.44	
**Vemurafenib +Cobimetinib**	Log-normal	meanlog	2.44	0.07	1380.64	3.24	0.08	1035.64
		sdlog	0.99	0.05		1.00	0.07	
	Log-logistic	shape	1.72	0.10	1385.78	1.76	0.14	1039.24
		scale	11.23	0.74		24.99	1.87	
	Generalized Gamma	mu	2.21	0.13	1378.73	3.16	0.18	1037.36
		sigma	1.01	0.05		1.06	0.13	
		Q	-0.52	0.26		-0.25	0.47	
	Log-logistic MCM	theta	0.18	NA	1349.99	0.33	NA	996.31
		shape	2.12	0.20		2.13	0.34	
		scale	9.20	0.80		19.23	4.71	
	Lognormal MCM	theta	0.16	NA	1348.06	0.17	NA	994.48
		meanlog	2.24	0.11		3.20	0.46	
		sdlog	0.83	0.08		0.92	0.20	
	Generalized Gamma MCM	theta	0.12	NA	1349.75	0.00	NA	996.22
		mu	2.22	0.13		3.29	0.23	
		sigma	0.90	0.17		1.10	0.19	
		Q	-0.26	0.51		-0.37	0.61	
	Spline-based Model	gamma0	-5.74	0.58	1379.64	-7.68	1.16	1037.08
		gamma1	3.17	0.43		3.15	0.71	
		gamma2	0.41	0.08		5.77	2.36	

Abbreviation: MCM: mixture cure model

Notes: Based on AIC and virtually inspection of fitted survival model, the following survival distributions were used for extrapolating long-term survival in base case analysis:

1. For triplet therapy, generalized gamma MCM was used for PFS and lognormal MCM for OS
2. For doublet therapy, lognormal MCM was used for both PFS and OS

© 2021 Cai C et al. *JAMA Network Open.*

eTable 2. Drug Dose and Costs Inputs

Drug	Dose	Monthly cost, $	Source
Atezolizumab	840 mg for days 1& 15	15 909	[1]
Vemurafenib	720 mg BID for 28 days for triplet regimen<br><br>960 mg BID for 28 days doublet regimen	10 586<br><br>14 115	[1]
Cobimetinib	60 mg QD for 21 days	8 999	[1]

Note: monthly costs were year 2020 adjusted.

eTable 3. Utilities Inputs

State	Utility	Source
PF	0.88	[2]
PP	0.52	[2]

Abbreviations: PF, progression-free health state; PP, post-progression health state

eTable 4. Adverse Events Rates and Costs Inputs

AE	Event rate (triplet regimen)	Event rate (doublet regimen)	Cost of treating AE	Cost Year	2020 Cost of treating AE	Triplet regimen cost, 2020 US$	Doublet regimen cost, 2020 US$	Source
Blood CPK increased	0.20	0.15	5583	2017	6094	1219	914	[3]
Rash	0.09	0.09	51	2015	59	5	5	[4]
Diarrhoea	0.02	0.03	775	2011	1005	20	30	[5]
Arthralgia	0.03	0.02	1947	2011	2524	76	50	[5]
Pyrexia	0.01	0.01	3304	2011	4283	43	43	[5]
ALT increased	0.13	0.09	5583	2017	6094	792	548	[3]
Lipase increased	0.20	0.21	5583	2017	6094	1219	1280	[3]
Aminotransferase increased	0.08	0.04	5583	2017	6094	488	244	[3]
Fatigue	0.01	0.00	2069	2011	2682	27	0	[5]
Nausea	0.00	0.02	1442	2011	1869	0	37	[5]
Pruritus	0.01	0.00	6914	2015	8030	80	0	[4]
Myalgia	0.01	0.00	1947	2011	2524	25	0	[5]
Photosensitivity reaction	0.01	0.03	5104	2015	5928	59	178	[4]
Maculopapular rash	0.13	0.10	51	2015	59	8	6	[4]
Amylase increased	0.10	0.16	5583	2017	6094	609	975	[3]
Hyperthyroidism	0.01	0.00	691	2015	803	8	0	[4]
Hypothyroidism	0.00	0.00	691	2015	803	0	0	[4]
Asthenia	0.02	0.01	8244	2017	8999	180	90	[3]
Blood creatinine increased	0.00	0.00	5583	2017	6094	0	0	[3]
Chorioretinopathy	0.00	0.00	5104	2015	5928	0	0	[4]
Blood ALK increased	0.02	0.01	5583	2017	6094	122	61	[3]

© 2021 Cai C et al. *JAMA Network Open.*

Dermatitis acneiform	0.02	0.02	51	2015	59	1	1	[4]
Vomiting	0.01	0.02	1442	2011	1869	19	37	[5]
Anaemia	0.01	0.03	851	2011	1103	11	33	[5]
Erythema	0.00	0.00	6914	2015	8030	0	0	[4]
Peripheral oedema	0.00	0.00	8255	2012	10 323	0	0	[6]
Sunburn	0.00	0.00	8255	2012	10 323	0	0	[6]
Decreased appetite	0.00	0.01	16 864	2012	21 089	0	211	[6]
Blood bilirubin increased	0.01	0.00	5583	2017	6094	61	0	[3]
Dry skin	0.00	0.00	8255	2012	10 323	0	0	[6]
Pneumonitis	0.01	0.00	13 282	2015	15 426	20	0	[4]

Abbreviations: AE, adverse events; ALT, alanine transaminase; ALP, alkaline phosphatase; CPK, creatine Phosphokinase

eTable 5. Parameter Input in Probabilistic Sensitivity Analysis

Variable	Base	Distribution
PFS cure rate parameter: triplet regimen	0.06	Binomial (0.06, 256)
PFS location parameter: triplet regimen	2.18	Lognormal (mean= 2.18, sd=4)
PFS scale parameter: triplet regimen	1.22	Lognormal (mean= 1.22, sd=5.6)
PFS cure rate parameter: doublet regimen	0.16	Binomial (0.16,258)
PFS location parameter: doublet regimen	2.24	Lognormal (mean=2.24, sd=1.77)
PFS shape parameter: doublet regimen	0.83	Lognormal (mean= 0.83, sd=1.28)
OS cure rate parameter: triplet regimen	0.55	Binomial (0.55,256)
OS location parameter: triplet regimen	2.72	Lognormal (mean= 2.72, sd=5.30)
OS scale parameter: triplet regimen	0.82	Lognormal (mean= 0.82, sd=2.73)
OS cure rate parameter: doublet regimen	0.17	Binomial (0.17,258)
OS location parameter: doublet regimen	3.20	Lognormal (mean= 3.20, sd=7.39)
OS scale parameter: doublet regimen	0.92	Lognormal (mean= 0.92, sd=3.21)
Cobimetinib cost per month	8999	Gamma (8999, 0.1889)
Vemurafenib (960mg) cost per month	14115	Gamma (14115, 0.201)
Atezolizumab cost per month	15909	Gamma (15909, 0.1999)
PPS cost per month	1316	Gamma (1316,0.2)
PPS utility	0.52	Lognormal (mean = 0.52, sd = 0.04)

Abbreviations: PFS, progression-free survival; OS, overall survival; sd, standard deviation

© 2021 Cai C et al. *JAMA Network Open.*

eReferences

1. Abaloparatide Ai. RED BOOK Online. Micromedex Healthcare Series [Database Online]. Truven Health Analytics; 2020.
2. Curl P, Vujic I, van't Veer LJ, Ortiz-Urda S, Kahn JG. Cost-effectiveness of treatment strategies for BRAF-mutated metastatic melanoma. PloS one. 2014;9(9):e107255.
3. Stellato D, Gerbasi ME, Ndife B, Ghate SR, Moynahan A, Mishra D, et al. Budget impact of dabrafenib and trametinib in combination as adjuvant treatment of BRAF V600E/K mutation-positive melanoma from a US commercial payer perspective. Journal of managed care & specialty pharmacy. 2019;25(11):1227-37.
4. Wong W, Yim YM, Kim A, Cloutier M, Gauthier-Loiselle M, Gagnon-Sanschagrin P, et al. Assessment of costs associated with adverse events in patients with cancer. PLoS One. 2018;13(4):e0196007.
5. Barzey V, Atkins MB, Garrison LP, Asukai Y, Kotapati S, Penrod JR. Ipilimumab in 2nd line treatment of patients with advanced melanoma: a cost-effectiveness analysis. Journal of medical economics. 2013;16(2):202-12.
6. Arondekar B, Curkendall S, Monberg M, Mirakhur B, Oglesby AK, Lenhart GM, et al. Economic burden associated with adverse events in patients with metastatic melanoma. Journal of managed care & specialty pharmacy. 2015;21(2):158-64.
7. Briggs A, Sculpher M, Claxton K. Decision modelling for health economic evaluation: Oup Oxford; 2006.

© 2021 Cai C et al. *JAMA Network Open.*
